# Domestic Correspondence

**Published:** 1891-03

**Authors:** C. S. Stockton

**Affiliations:** Newark


### Domestic Correspondence.

TO THE EDITOR:

SIR,—Please allow me a word in regard to the electric deposit-plate. Mr. E. E. Clark, the owner and manager, has been absent in the West in its interest most of the year, and I have, in his absence, undertaken to look after the making of the plates. For a considerable time past, the plates have been sadly deficient in gold, sometimes not enough being put on to properly vulcanize over.

I was unable to account for it, knowing that full quantity of gold was supplied. The secret has been discovered, and the person accused of using the gold furnished to him for coating the plates is now in jail awaiting trial for larceny.

The plates as now made are all right in every respect, and it is hoped that all its former friends will again come back to its use.

Yours, etc.,

C. S. STOCKTON.

NEWARK, November 17, 1890.

